# The deltoid ligament is constantly formed by four fascicles reaching the navicular, spring ligament complex, calcaneus and talus

**DOI:** 10.1002/ksa.12173

**Published:** 2024-05-17

**Authors:** Miki Dalmau‐Pastor, Francesc Malagelada, Matteo Guelfi, Gino Kerkhoffs, Jon Karlsson, James Calder, Jordi Vega

**Affiliations:** ^1^ Human Anatomy and Embryology Unit, Department of Pathology and Experimental Therapeutics University of Barcelona Barcelona Spain; ^2^ MIFAS By GRECMIP (Minimally Invasive Foot and Ankle Society) Merignac France; ^3^ Foot and Ankle Unit, The Royal London Hospital Barts Health NHS Trust London UK; ^4^ Foot and Ankle Unit, Casa di Cura Villa Montallegro Genoa Italy; ^5^ Department of Orthopaedic Surgery “Gruppo Policlinico Di Monza” Clinica Salus Alessandria Italy; ^6^ Department of Orthopedic Surgery and Sports Medicine Amsterdam UMC location University of Amsterdam Amsterdam The Netherlands; ^7^ Amsterdam Movement Sciences Amsterdam The Netherlands; ^8^ Amsterdam Collaboration for Health & Safety in Sports (ACHSS) International Olympic Committee (IOC) Research Center Amsterdam UMC Amsterdam The Netherlands; ^9^ Department of Orthopaedics, Sahlgrenska Academy University of Gothenburg Gothenburg Sweden; ^10^ Fortius Clinic London UK; ^11^ iMove Traumatology Tres Torres Barcelona Spain

**Keywords:** anatomy, ankle, ankle instability, ankle ligaments

## Abstract

**Purpose:**

The medial collateral ligament of the ankle, or deltoid ligament, can be injured in up to 40% of patients who sustain an ankle inversion sprain. Reporting injuries of the deltoid ligament is not easy due to confusion in the current anatomical descriptions, with up to 16 fascicles described, with variable frequencies. The purpose of this study was to clarify the anatomy of the deltoid ligament.

**Methods:**

Thirty‐two fresh‐frozen ankle specimens were used for this study. Careful dissection was undergone until full visualization of the deltoid ligament was achieved and measurements taken.

**Results:**

The deltoid ligament was found to have four constant fascicles in two layers. The superficial layer consists of the tibionavicular, tibiospring and tibiocalcaneal fascicles, while the deep layer consists of the tibiotalar fascicle. Measurements of these fascicles are given in detail. The tibiotalar fascicle and the anterior part of the tibionavicular fascicle were found to be intra‐articular structures.

**Conclusion:**

The deltoid ligament has a constant number of fascicles divided into a superficial and a deep layer. This clarification of the anatomy and terminology of the deltoid ligament and its fascicles will help clinical view, diagnosis and (interdoctor)communication and treatment. The ligamentous fibres of the deep layer, as well as the anterior fibres of the superficial layer (tibionavicular fascicle) are intra‐articular, which could negatively impact its healing capacity, explaining chronicity of these types of injuries.

**Level of Evidence:**

Not applicable (cadaveric study).

**Abbreviations**
AntC: anterior colliculus
ATFL: anterior talofibular ligament
CAI: chronic ankle instability
CFL: calcaneofibular ligament
IRB: institutional review board
PostC: posterior colliculus
Pre‐C: anterior part of the anterior colliculus
PTFL: posterior talofibular ligament
TC: TibioCalcaneal fascicle
TN: TibioNavicular fascicle
TS: TibioSpring fascicle

## INTRODUCTION

Ankle sprains are one of the most common injuries amongst the general population and during sports practice. Most sprains are inversion/supination sprains and therefore mainly affect the lateral collateral ligament of the ankle, which is formed by the anterior talofibular (ATFL), the calcaneofibular (CFL) and the posterior talofibular ligaments (PTFL). It is generally considered that the ATFL, specifically its intra‐articular superior fascicle [6], may sustain at least some degree of injury in all ankle sprains. The inferior fascicle of the ATFL, which is connected with the CFL [22], is the next structure to become injured. In cases of moderate to severe inversion sprains, the CFL is also injured.

Finally, the PTFL would rarely get injured during ankle sprains.

While most patients suffering an ankle sprain are treated nonsurgically, it is reported that between 30% and 40% of them will suffer from chronic pain because of that sprain [7] and up to 33% will develop chronic ankle instability (CAI) associated with a feeling of subjective instability and recurrent sprains [19].

Classically, it has been estimated that up to 40% of those patients will either have or develop an injury of the medial collateral ligament (or the deltoid ligament) [9]. However, accurately reporting deltoid injuries is not easy, due to the wide variety of anatomical descriptions that can be found in the literature [1, 4, 8, 10, 12, 13, 16, 23, 24, 25]. The most accepted description of the deltoid ligament divides it in up to six fascicles distributed into two layers (superficial and deep) [14]. Logically, the long fibres of the ligament are in the superficial layer and the short fibres are in the deep layer. Normally four fascicles of the superficial layer of the deltoid ligament are described: tibionavicular and tibiospring, present in every case and superficial tibiotalar and tibiocalcaneal, nonconstant fascicles. In the deep layer, two fascicles are commonly described: posterior tibiotalar, always present, and deep anterior tibiotalar, nonconstant. However, a variable number of fascicles (with varying frequencies of appearance) have been reported in anatomical studies. Some authors stick to six fascicles; [1, 2, 8, 24] Cromeens et al. added to that description the ‘tibiocalcaneonavicular’, the ‘superficial posterior tibiotalar’, the ‘deep posterior tibiotalar’ and the ‘inferoplantar longitudinal ligaments’ [4]. Panchani et al. described the ‘band deep to tibiocalcaneal ligament’ and ‘band posterior to sustentaculum tali’ [16]. Zamperetti et al. added an accessory bundle, the ‘deep intermedial tibiotalar ligament’ [25]. Some of these fascicles could be the one Rasmussen named ‘intermediate tibiotalar ligament’ in 1983 [18], as they all appear to be located deep into the tibiocalcaneal fascicle, but this has not been reported in earlier studies. More recently, Ismail et al. [11]. published an anatomical study, adding the ‘tibiotalocalcaneal ligament’ as a constant fascicle, and the ‘anterior tibiotalonavicular ligament’ was shown to be present in 60% of cases, together with four of the formerly mentioned constant fascicles.

It is therefore apparent that the current description of the deltoid ligament is confusing, with a total of 16 different fascicles reported in the literature. This has created problems for the clinicians when reporting injuries of this structure and becomes a problem for repair and reconstruction surgical procedures, as the lack of a detailed description leads to uncertainty in terms of which exact part of the ligament is being repaired/reconstructed, and it also makes comparisons between studies difficult. A review of the literature demonstrates how most of the anatomical papers studying the deltoid ligament do so from only an extra‐articular view and do not study the structure from an intra‐articular view of the ligament [1, 2, 4, 8, 10, 16, 18, 23, 25]. In a previous study on the lateral ankle ligaments, an intra‐articular view helped the authors to describe new and previously unknown connections between its components [5], and we believe that from an anatomical point of view, it is necessary to study ligaments from both aspects (extra and intra‐articular). In words of the 19th‐century anatomist Barclay‐Smith: ‘In dissecting the ligaments about a joint, in order to determine their disposition, it is important to do so from every aspect, and not only to examine them superficially, but also, when possible, to investigate the surface they present to the joint‐cavity’ [20]. To the best of our knowledge, the most recent study using an intra‐articular view to study the deltoid ligament dates from 1979 [17].

For the above‐mentioned reasons, we claim that the current anatomical descriptions of the deltoid ligament are confusing and not helpful enough for clinicians. In addition, previous anatomical studies have focused on describing fascicles that sometimes are difficult to find. Moreover, we claim that the deltoid ligament can be described more consistently when using both an inside and outside view. As a result, clinicians are helped in their clinical view and examination enabling better diagnosis and consequently better (interdoctor)communication and treatment.

To facilitate this interdisciplinary and intradisciplinary communication and further clarify deltoid ligament anatomy, we propose a simpler anatomical description for this structure.

It should allow a clear and simple depiction of the anatomy of the deltoid ligament, enabling physicians to establish a clear diagnosis and subsequent indication(s) for surgical repair or reconstruction.

## MATERIALS AND METHODS

This study was conducted at the dissection room of the University of Barcelona, Bellvitge Campus. Ethical permission was granted to conduct anatomical studies on human samples, with IRB00003099.

Thirty‐two fresh‐frozen below‐the‐knee specimens were used for this study. Sample calculation was not performed, as the maximum numbers of cadaveric specimens available were used for the study.

As it is well recognized that the deltoid ligament has fibres distributed in two anatomical planes (superficial and deep), it was deemed necessary to observe the superficial layer of the deltoid ligament from a medial view and to observe the deep layer from a lateral (intra‐articular) view.

**Figure 1:**
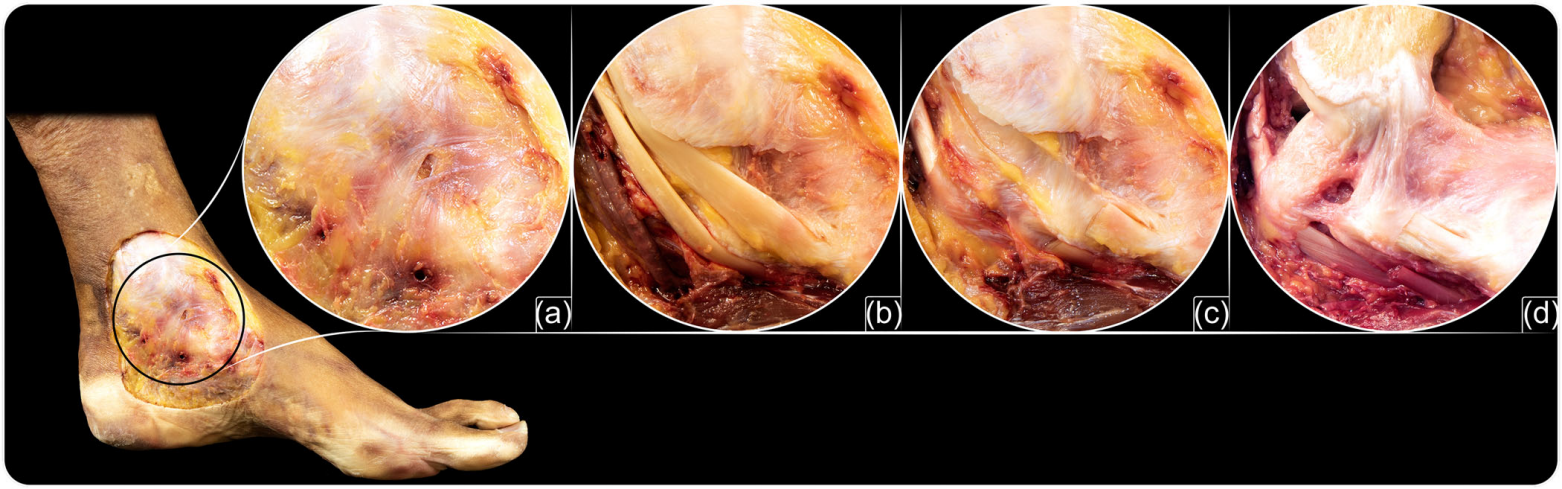
Methodology used to dissect the superficial layer of the deltoid ligament. (a) Step 1: A skin window on the medial aspect of the ankle was performed to observe the flexor retinaculum. (b) Step 2: The flexor retinaculum was opened to observe the tibialis posterior and flexor digitorum longus tendons. (c) Step 3: Both tendons were resected. (d) Step 4: With extreme care, the synovial sheath of both tendons was resected from the superficial layer of the deltoid ligament, without disrupting any of the fascicles of the ligament.

**Figure 2:**
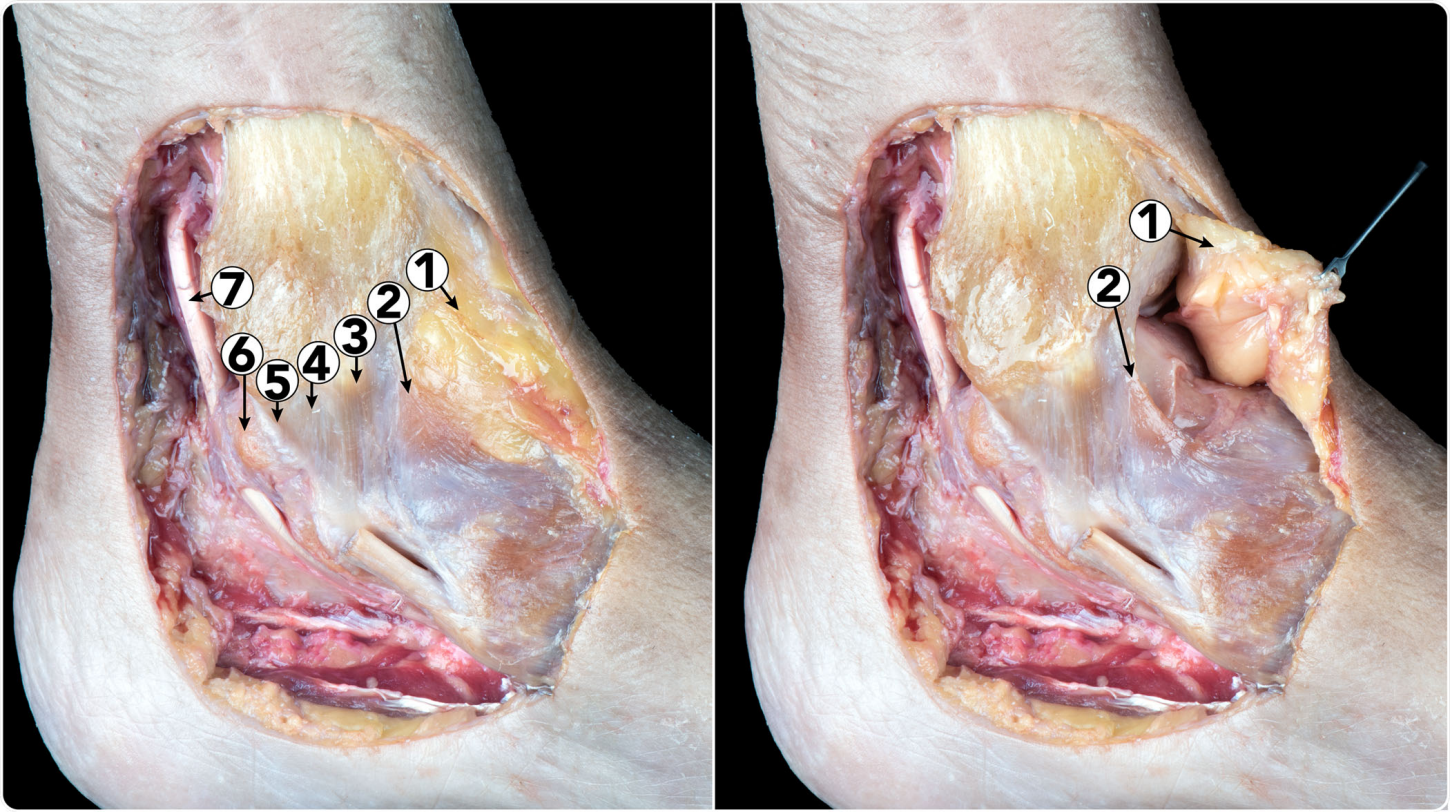
Dissection showing the relation between the anterior ankle joint capsule and the anterior part of the deltoid ligament. 1. Anterior ankle joint capsule. 2. Tibionavicular fascicle of the deltoid ligament. 3. Tibiospring fascicle of the deltoid ligament. 4. Tibiocalcaneal fascicle of the deltoid ligament. 5. Posterior part of the tibiotalar fascicle (deep layer) of the deltoid ligament. 6. Posteromedial talar tubercle. 7. Flexor hallucis longus tendon.

**Figure 3:**
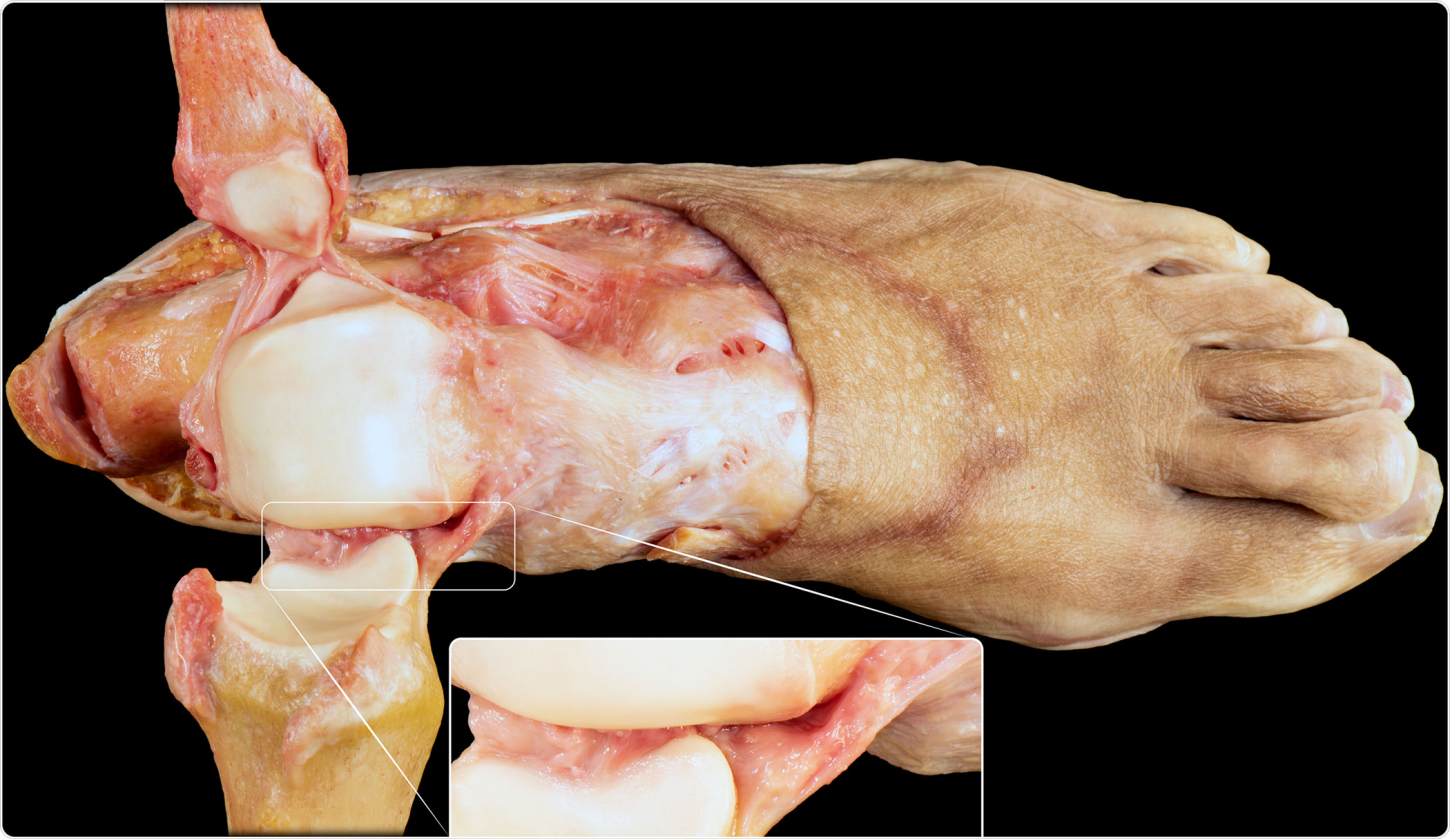
Figure demonstrating step 8 of the methodology: dislocation of the tibiofibular syndesmosis to observe the deep layer of the deltoid ligament.

The plane‐per‐plane dissection was performed by an experienced anatomist following these steps:

1. A skin window on the medial aspect of the ankle was performed to observe the flexor retinaculum (Figure 1a).
2. The flexor retinaculum was opened to observe the tibialis posterior and flexor digitorum longus tendons (Figure 1b).
3. Both tendons were resected (Figure 1c).
4. With extreme care, the synovial sheath of both tendons was resected from the superficial layer of the deltoid ligament, without disrupting any of the fascicles of the ligament. The sustentaculum tali was identified during this step (Figure 1d).
5. The tendons of the anterior compartment of the leg were exposed and resected (tibialis anterior, extensor hallucis longus, extensor digitorum longus, peroneus tertius). The anterior capsule of the ankle was exposed.
6. With extreme care, dissection of the ankle capsule was performed, paying attention not to affect the anterior fascicles of the deltoid ligament (Figure 2).
7. Measurements of the superficial layer of the deltoid were taken.
8. The tibiofibular syndesmosis was dislocated through resection of the anterior tibiofibular ligament, posterior tibiofibular ligament, interosseous ligament and interosseous membrane. The tibia was then mobilized medially to allow observation of the deep layer of the deltoid ligament (Figure 3).
9. Synovium was carefully resected from the deep layer of the deltoid ligament.
10. Measurements of the deep layer of the deltoid ligament were taken.

Steps 3 and 5 are critical. An over‐dissection in these steps could end with a resection of some of the fascicles of the ligament (most likely of the tibiocalcaneal in step 3 or tibionavicular fascicles in step 5), therefore modifying their frequency appearance, size or morphology. In addition, an effort was made not to over‐dissect the ligament, avoiding separation, for example, of the posterior tibiotalar fibres in two different fascicles.

### Statistical analysis

Results were obtained using the statistical programme SPSS version 19 (IBM). Quantitative variables were expressed as means, median, standard deviation and ranges. The Shapiro–Wilk normality test was used to study for normal distribution of each of the variables. A two‐tailed paired *t* student test was used to compare attachments within each fascicle. Correlation analysis was used to describe the strength (degree) and the direction of the relationship between variables. Pearson and Spearman correlation coefficients were used according to the distribution type for each variable. Statistical significance was set at a *p* value of <0.05.

## RESULTS

**Figure 4:**
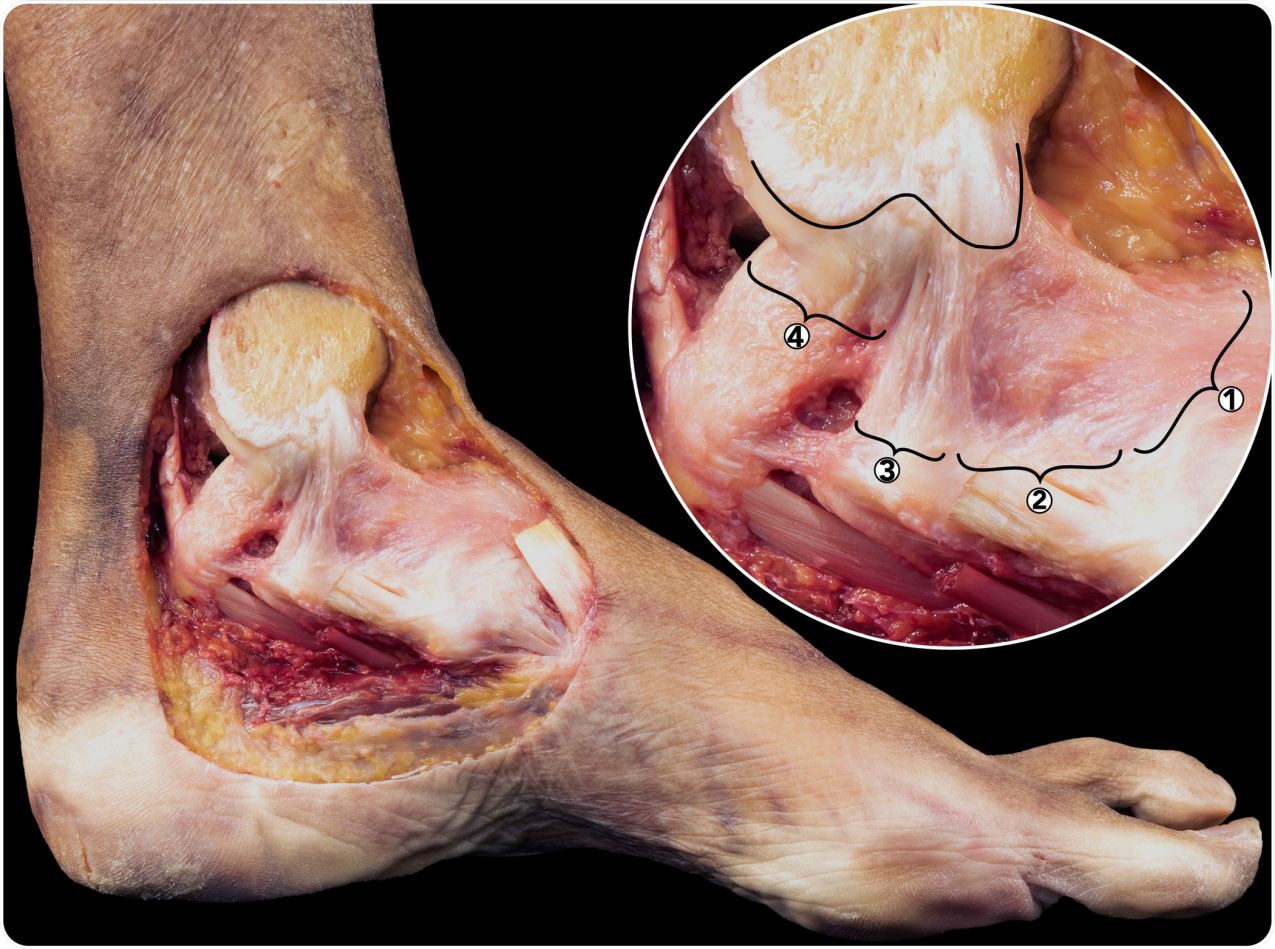
Dissection showing the deltoid ligament. The anterior and posterior colliculus have been outlined. 1. Tibionavicular fascicle. 2. Tibiospring fascicle. 3. Tibiocalcaneal fascicle. 4. Posterior part of the tibiotalar fascicle (deep deltoid).

The deltoid ligament was found to have four different fascicles in two layers: three superficial fascicles and one deep fascicle (Figure 4). The superficial layer had the longest fibres of the deltoid ligament corresponding to the tibionavicular, tibiospring and tibiocalcaneal fascicles; the deep layer had the shortest fibres of the deltoid ligament corresponding to the tibiotalar fascicle.

**Figure 5:**
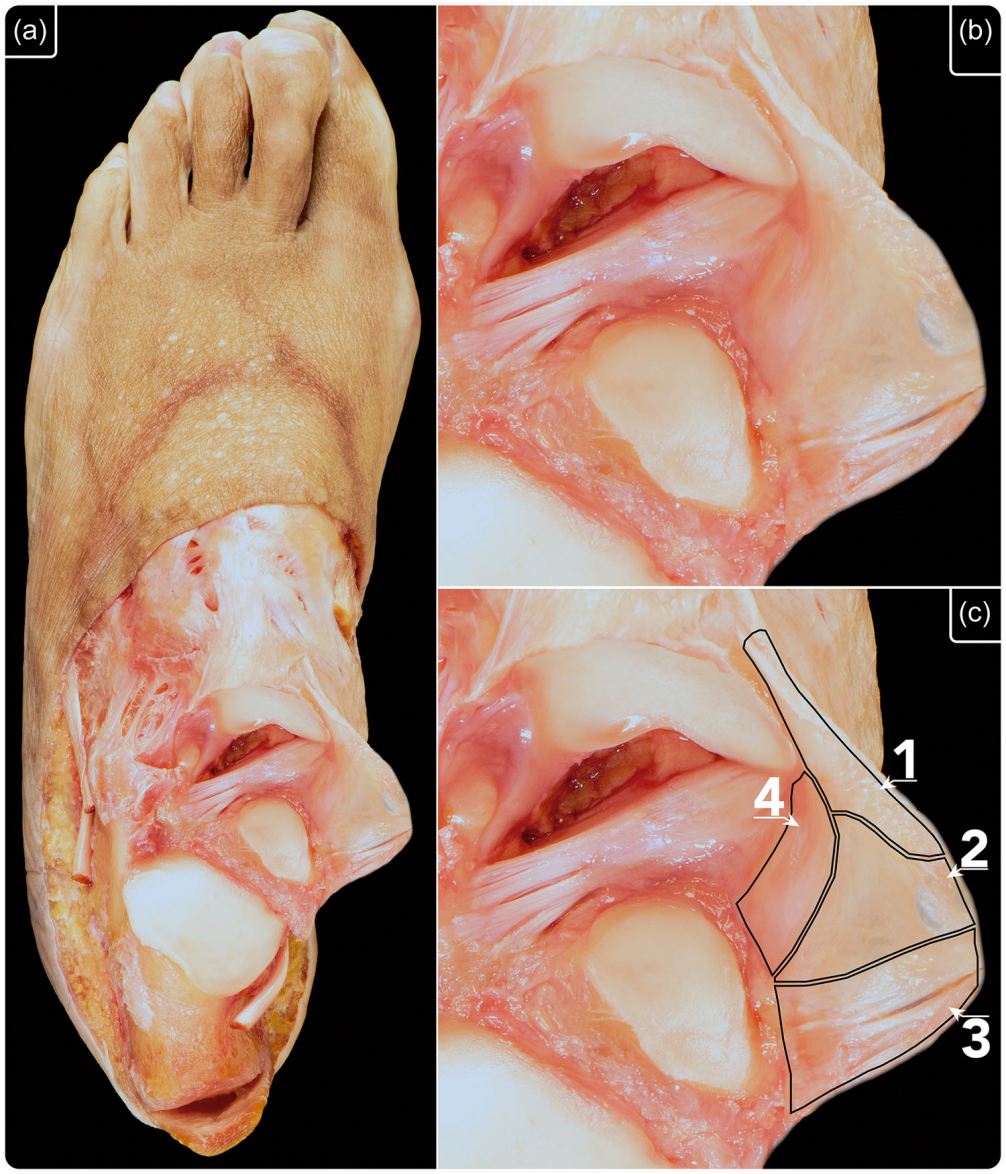
Dissection demonstrating the anatomy of the superficial deltoid (a). The talus has been removed to show the insertions of the fascicles of the superficial layer of the deltoid ligament, visible in macrophotography (b) and then outlined (c). 1. Tibionavicular fascicle. 2. Tibiospring fascicle. 3. Tibiocalcaneal fascicle. 4. Superomedial calcaneonavicular ligament (part of the spring ligament complex, covered by fibrocartilage).

**Figure 6:**
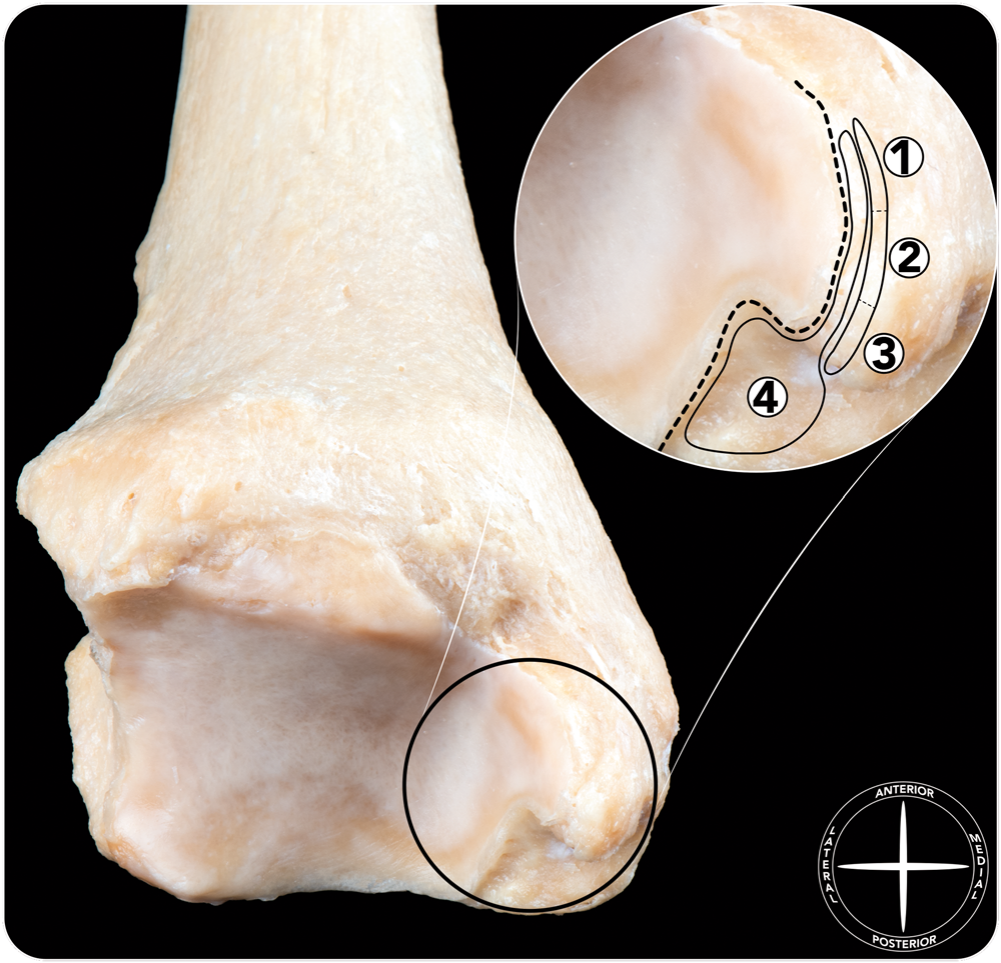
Anteroinferior view of a tibia, where the medial limit of the articular cartilage (discontinuous line) and the insertion of the superficial and deep deltoid have been outlined. 1. Tibionavicular fascicle. 2. Tibiospring fascicle. 3. Tibiocalcaneal fascicle. 4. Tibiotalar fascicle.

**Figure 7:**
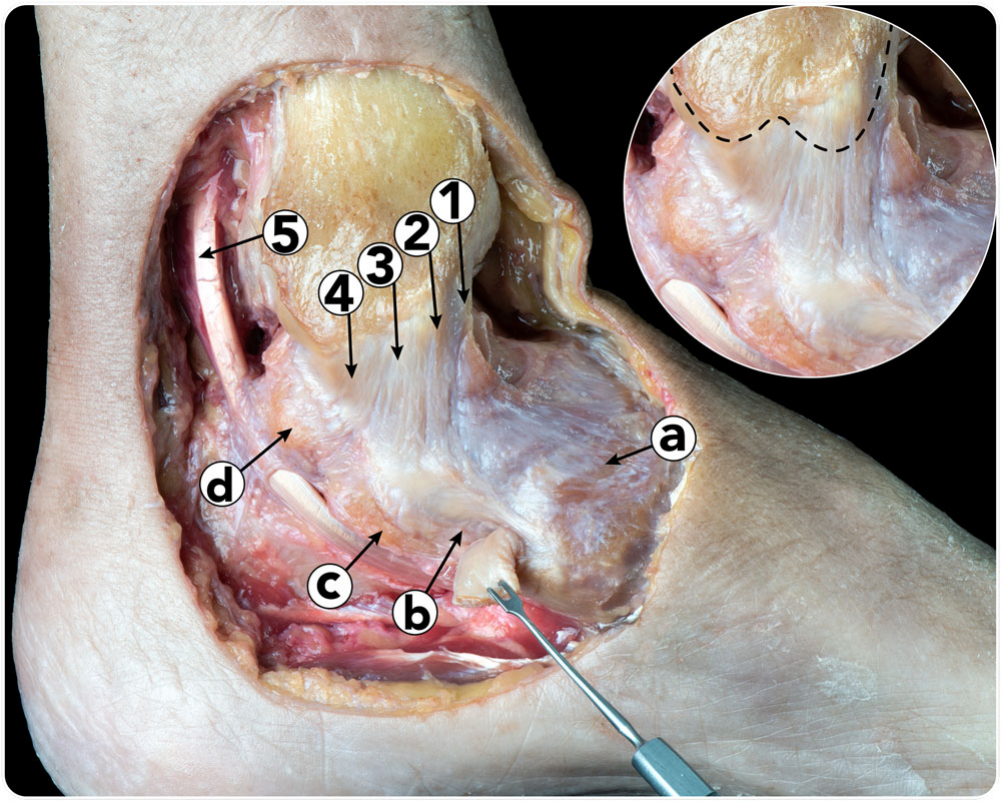
Figure highlighting the insertion points visible when observing the deltoid ligament superficially. (a) Navicular. (b) Superomedial calcaneonavicular ligament (part of the spring ligament complex; fibrocartilage visible due to retraction of tibialis posterior tendon). (c) Sustentaculum tali. (d) Posteromedial talar tubercle. 1. Tibionavicular fascicle. 2. Tibiospring fascicle. 3. Tibiocalcaneal fascicle. 4. Posterior part of the tibiotalar fascicle. 5. Flexor hallucis longus tendon.

The insertions of the fascicles were located as follows:

- Superficial layer (Figure 5): the proximal insertions were located on the medial (extra‐articular) part of the medial malleolus.
  - Tibionavicular: from the anterior part of the anterior colliculus to the superior and medial part of the navicular. The anterior part of this fascicle was found to be intra‐articular.
  - Tibiospring: from the middle part of the anterior colliculus to the superior margin of the superomedial calcaneonavicular ligament.
  - Tibiocalcaneal: from the posterior part of the anterior colliculus to the superior margin of the sustentaculum tali.

- Deep layer: the proximal insertions were located on the lateral (intra‐articular) part of the medial malleolus, just medial to the articular cartilage (Figure 6).
  - Tibiotalar: these are intra‐articular fibres, originating from the lateral side of the anterior colliculus and medial and lateral side of the posterior colliculus to the talus, with its insertion occupying the whole extent of the bone under the medial comma‐shaped articular surface of the talus. According to this description, the superficial fascicles (tibionavicular, tibiospring, tibiocalcaneal) occupy just the anterior colliculus of the medial malleolus, with the deep fascicle (tibiotalar) being visible just posterior to the tibiocalcaneal fascicle (most posterior fascicle of the superficial layer). When visualizing a dissection of the superficial deltoid, fibres can be seen reaching four insertion points: navicular, spring ligament complex and calcaneus for the superficial layer, but also the most posterior part of the deep deltoid is visible, with its most superficial insertion on the posteromedial talar tubercle (Figure 7). For the superficial deltoid measurements, we found that for every fascicle (Tibionavicular, Tibiospring and Tibiocalcaneal) the distal attachment proved to be wider than the proximal one in all cases, and this is also reflected in the mean values (p < 0.05) (Table 1). Regarding the deep deltoid measurements, ligament 209 fibres originated from the anterior and posterior colliculus. Both fascicles were located intra‐articularly and appeared as straight bands with almost equal widths proximally and distally in their attachments. However, the most anterior part (anterior to the anterior colliculus) also showed wider distal attachments than proximal, featuring a fan‐shaped anatomy that flares distally (p < 0.05). Measurements of the deep deltoid were taken dividing its tibial origin in: Pre‐C (anterior part of the anterior colliculus), AntC (anterior colliculus) and PostC (Posterior colliculus) (Table 2).

**Table 1:** Measurements and comparisons of proximal and distal attachments of the superficial deltoid and its fascicles.

	TN Prox	TN Dist	TS Prox	TS Dist	TC Prox	TC Dist
Mean	4.10	14.90	5.30	7.86	6.31	9.83
Median	3.98	14.91	5.25	7.98	6.09	9.98
SD	1.17	3.46	1.61	2.16	1.24	2.54
Min	2.16	7.27	1.73	2.88	4.25	2.36
Max	6.53	24.03	7.79	12.6	9.51	15.29
*p* value	0.0001		0.0001		0.0001	
CI	−12.068 to −9.528		−3.164 to −1.962		−4.510 to −2.533	

Abbreviations: TC, tibiocalcaneal fascicle; TN, tibionavicular fascicle; TS, tibiospring fascicle.

**Table 2:** Measurements and comparisons of proximal and distal attachments of the deep deltoid and its fascicles.

	Pre‐C Prox	Pre‐C Dist	AntC Prox	AntC Dist	PostCProx	PostC Dist
Mean	5.98	10.29	9.67	8.84	9.66	10.48
Median	5.57	10.35	9.63	8.86	9.37	10.42
SD	1.54	1.68	1.79	1.53	1.24	1.59
Min	3.5	7.36	6.65	6.16	7.66	7.94
Max	8.99	13.9	14.55	11.79	12.43	13.54
*p* value	0.0001		0.0226		0.0118	
CI	−5.087 to −3.537		0.128 to 1.545		−1.445 to −0.199	

Abbreviations: AntC, anterior colliculus; PostC, posterior colliculus; PreC, anterior part of the Anterior colliculus.

Measurements of the deep deltoid were taken dividing its tibial origin according to its insertion on the anterior colliculus: anterior or pre‐collicular (anterior part of the anterior colliculus), intermediate or collicular (anterior colliculus) and posterior or post‐collicular (Posterior to the anterior colliculus).

**Figure 8:**
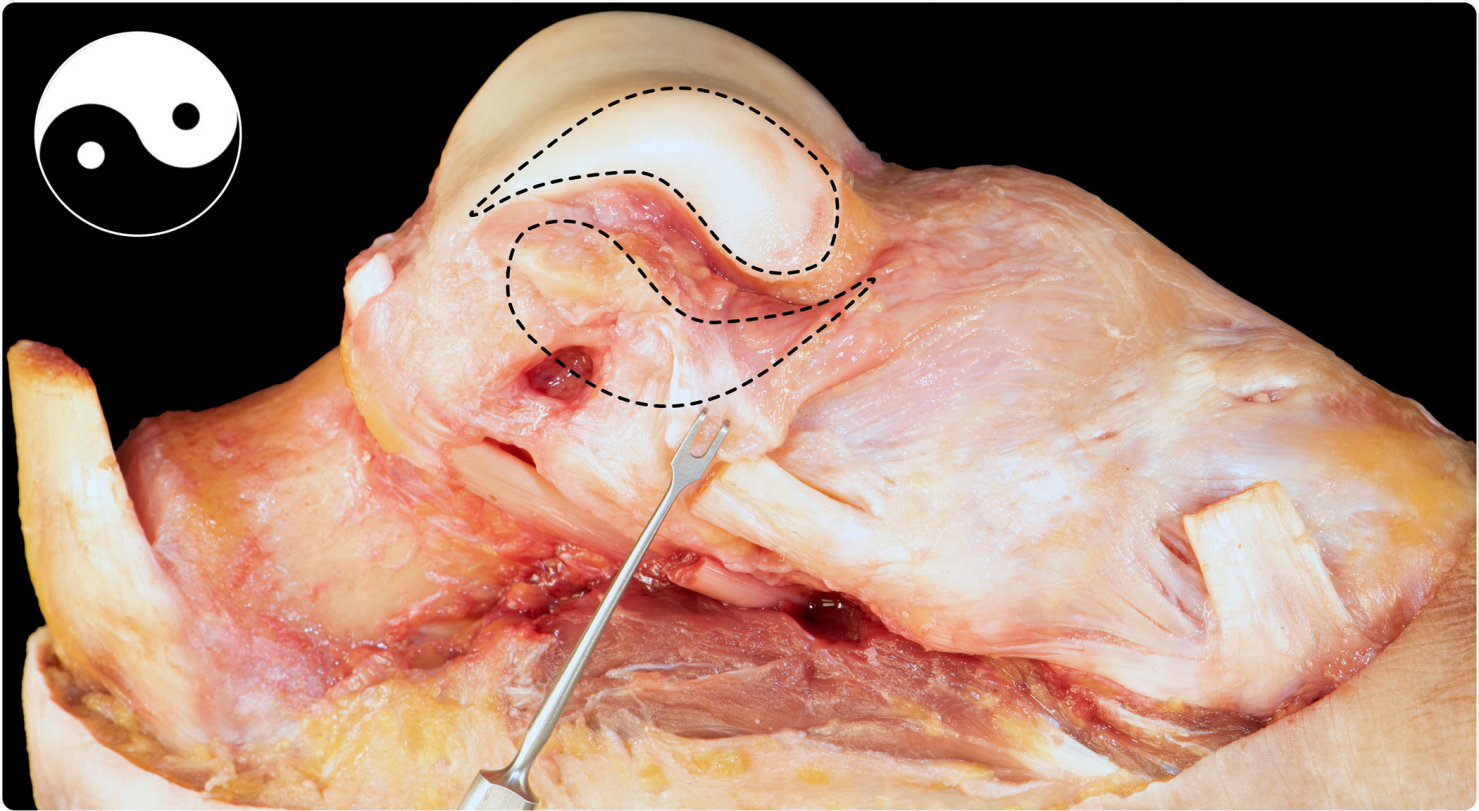
Dissection showing the talar insertion of the deep deltoid. The morphology of the medial talar articular surface (comma‐shaped) has been outlined. The talar insertion of the tibiotalar fascicle has an inverted comma morphology, thus resembling a yin‐and‐yang symbol.

When disarticulating the joint and observing the insertions of the ligament onto the bone, we observed that the footprint at the talus featured a comma‐shaped anatomy with a wider attachment posteriorly. The widest distance was posterior, and as it was progressing anteriorly, the ligament was thinning out. Similarly, but in the opposite direction, the medial articular surface of the talus was wider anteriorly and became thinner while progressing posteriorly. In fact, the insertion of the tibiotalar fascicle of the deltoid ligament on the talus resembles a ‘yin and yang’ fashion, with two comma‐shaped structures: one is the medial talar articular surface (wider anteriorly) and the other is the footprint of the tibiotalar fascicle (wider posteriorly) (Figure 8).

## DISCUSSION

The most important finding of this study is that the deltoid ligament was found to have a constant number of fascicles, attaching to the navicular, the spring ligament complex, the calcaneus and the talus. The tibiotalar fascicle (deep deltoid) and anterior part of the tibionavicular fascicle (superficial deltoid) were found to be in an intra‐articular position.

In the present study, the anatomy of the deltoid was interpreted from an anatomical and functional point of view, describing mainly the bones that are united by each type of fibres. This was done to avoid describing a high number of fascicles, as this might just be confusing and of no use to the physicians and orthopaedic surgeons involved in treating deltoid ligament injuries. In addition, we performed an intra‐articular observation of the deltoid ligament, allowing a description in detail of the deep layer of the ligament, which has not been performed in the modern anatomical studies. It is important to note that what is presented in this study is not entirely new; the first report of the deltoid ligament having these four fascicles (tibiotalar, tibionavicular, tibiospring and tibiocalcaneal) dates from 1956 [3] and as pointed out the most recent study looking at deltoid ligament anatomy from an intra‐articular view dates from 1979 [17]. However, after reviewing the literature and finding 16 different fascicles described within the same ligament, it became evident that the anatomical description of the deltoid ligament needed clarification to help with injury interpretation and surgical repair. In this study, we have described a superficial layer formed by three fascicles: tibionavicular, tibiospring and tibiocalcaneal; and a deep layer, formed solely by tibiotalar fibres (originated from the anterior and posterior colliculus).

The tibionavicular is the most anterior fascicle of the superficial deltoid. Its lateral margin is continuous with the anterior ankle joint capsule, and our description is similar to previous reports [11, 16, 24].

The tibiospring fascicle is found just posterior to the tibionavicular fascicle, also in the superficial layer of the deltoid. This fascicle inserts in the superomedial calcaneonavicular ligament and most studies agree on it being a constant fascicle [1, 2]. The tibiocalcaneal fascicle was found in every case, inserting to the sustentaculum tali of the calcaneus. In this, the present study differs from the literature, as the tibiocalcaneal fascicle has usually been described as a nonconstant fascicle [2, 8, 16]. During this study, we paid attention to this point, and realized that, when resecting the synovial sheath of tibialis posterior and flexor digitorum longus tendons, it was indeed very easy to inadvertently remove some of the fibres that form this tibiocalcaneal fascicle. We suspect this could partly explain why the tibiocalcaneal fascicle has been reported as a nonconstant structure while, as showed in this study, it is indeed a constant part of the deltoid ligament.

Finally, the deep layer of the deltoid ligament was investigated in this study without dissecting the superficial layer off the deep layer. Instead, the tibiofibular syndesmosis was opened to observe the deltoid ligament from an intra‐articular (lateral) view, which allowed for the description of the deep layer with the certainty of doing so without having disrupted any part of the ligament during the dissection process. As described in the results section, the tibiotalar fibres were found to insert in a ‘yin‐and‐yang’ fashion, as the morphology of the insertion displayed a comma‐shape, inverted with the comma‐shaped medial talar articular surface.

From a clinical perspective, it is important to remark the fact that the deep layer of the deltoid ligament is intraarticular, corresponding to the most anterior part of the tibionavicular fascicle (superficial deltoid). The posterior part of the tibionavicular fascicle, tibiospring fascicle and tibiocalcaneal fascicle are all extra‐articular structures. Following descriptions of the superior fascicle of ATFL as an intra‐articular extra synovial structure [6, 22] that synovial environment has explained the inability to heal naturally of the ATFL, resembling the anterior cruciate ligament of the knee [15]. This has also given rise to the concept of ankle microinstability. This nonhealing ATFL's superior fascicle could help understand the high percentage of patients suffering from chronic pain after an ankle sprain. Following an injury, the remnant of ATFL's superior fascicle is likely to resynovialize, and therefore healing cannot be achieved. Currently, no study confirms this hypothesis, but clinical studies certainly suggest that this is the case [21].

The current study shows that the deep deltoid and part of the anterior deltoid are also intra‐articular extra‐synovial structures. If we apply the same concept seen in the knee with the ACL, we can imagine the ligament remnant to resynovialize after rupture, hypothetically interrupting or hindering the normal healing process of the ligament. Nevertheless, attention must be put on the fact that distances between ligament insertions are much shorter in the ankle than in the knee, which could help the healing of ankle ligaments after injury. In addition, noncomplete ruptures of the ankle ligaments are common, and having some fibre continuity could also be important in ankle ligament healing.

The clinical implications of this study are clear. It is not possible for clinicians and surgeons to discuss and improve diagnosis and treatment if they are dealing with a structure with up to 16 fascicles, most of them nonconstant. The clarification here presented will help in having a common terminology and anatomical description, which should help in discussing clinical presentation and treatment options. Finally, the description of intra‐articular deltoid ligament fibres will help in understanding why some injuries of the deltoid (specially deep deltoid injuries) are more difficult to heal than others (superficial deltoid injuries).

This study has some limitations. One is inherent to any cadaveric study, where the difficulty in having human samples for studies makes that the validity of the study could be improved with a larger sample. For that reason, a power size calculation was not performed. Second, the art of anatomical plane‐by‐plane dissection is subject to interpretation, as demonstrated in the literature search on the deltoid ligament. However, this is also a strength of this article, where the dissections were carried out following a strict protocol by an experienced anatomist, which is likely to increase the validity of the study.

## CONCLUSION

The deltoid ligament, or medial collateral ligament of the ankle, is constantly formed by four fascicles in two layers. A superficial layer with the tibionavicular, tibiospring and tibiocalcaneal fascicles and also a deep layer with the tibiotalar fascicle. The anterior part of the tibionavicular fascicle and the tibiotalar fascicles are intra‐articular, but extrasynovial structures will likely have a negative impact on its healing capacity.

## AUTHOR CONTRIBUTIONS

**Miki Dalmau‐Pastor**: Conceptualization; methodology; writing. **Francesc Malagelada**: Methodology; editing; data analysis. **Matteo Guelfi**: Methodology; writing. **Gino Kerkhoffs**: Writing; editing. **Jon Karlsson**: Writing; editing. **James Calder**: Writing; editing. **Jordi Vega**: Conceptualization; methodology; writing.

## CONFLICT OF INTEREST STATEMENT

The authors declare no conflict of interest.

## ETHICS STATEMENT

Ethics permission was obtained as disclosed in the methodology of the manuscript (IRB00003099).
